# Suspension-Adapted HEK293FT Cells Enable High-Density Transfection for Efficient Lentiviral Vector Production in CAR-T Therapy

**DOI:** 10.3390/bioengineering13080856

**Published:** 2026-07-24

**Authors:** Alexandr Shevtsov, Aitolkyn Kydyrbayeva, Gaziza Nigmatulla, Viktoriya Keyer, Tolganay Kulatay, Gulzat Zauatbayeva, Bakytkali Ingirbay, Maral Zhumabekova, Dinara Zharlyganova, Alexandr V. Shustov

**Affiliations:** National Center for Biotechnology, Korgalzhin hwy 13/5, 010000 Astana, Kazakhstan; shevtsov@biocenter.kz (A.S.); aitolkyn.yk@gmail.com (A.K.); nigmatullag@gmail.com (G.N.); keer@biocenter.kz (V.K.); kulatay@biocenter.kz (T.K.); zauatbaeva@biocenter.kz (G.Z.); ingirbay@biocenter.kz (B.I.); zhumabekova@biocenter.kz (M.Z.); dinarazh@mail.ru (D.Z.)

**Keywords:** lentiviral vectors, HEK293FT cells, suspension adaptation, high-density transfection, PEI transfection, serum-free medium, CAR-T therapy, transient transfection, cell line development, viral vector manufacturing

## Abstract

Lentiviral vector (LV) production for CAR-T therapy remains challenging due to the limited scalability of adherent HEK293 cell cultures. To address this, we adapted HEK293FT cells directly to serum-free FreeStyle 293 Expression Medium, generating a novel suspension cell line, HEK293FT-DS. We characterized growth kinetics, stability over 35 passages, and transient transfection parameters (PEI:DNA ratio, cell density). The suspension-adapted cells grew with >93% viability and a specific growth rate of 0.54 day^−1^, and maintained stable viable cell density (1.51 ± 0.03 × 10^6^ cells/mL) over 35 passages. The optimal PEI:DNA ratio was 2.5:1, and increasing the transfection cell density to 16 × 10^6^ cells/mL boosted functional titers to approximately 9 × 10^6^ TU/mL in clarified supernatant. Scaled production (1 L culture supernatant per batch) yielded up to 5 × 10^9^ TU. The resulting vectors efficiently transduced primary human T cells (57% CAR-positive cells) in a CliniMACS Prodigy-based process. The platform based on suspension-adapted HEK293FT-DS cells enables high-density transfection and provides a cost-effective, scalable alternative to commercial LV production systems, particularly suited for academic CAR-T cell manufacturing.

## 1. Introduction

Chimeric antigen receptor (CAR) T-cell therapy has revolutionized the treatment of hematological malignancies, demonstrating remarkable clinical efficacy against leukemias, lymphomas, and multiple myeloma [1,2]. Since the first FDA approval in 2017, numerous CAR-T products have either received regulatory approval or are in late-stage clinical trials—a testament to the maturity of this therapeutic technology [3]. Yet approval of commercial CAR-T therapies such as Kymriah and Yescarta has not guaranteed widespread access, because their high cost remains a formidable barrier to broad adoption, particularly in developing economies. A substantial portion of the total price of CAR-T therapy is attributable to the high cost of clinical-grade lentiviral (LV) and gamma-retroviral (gRV) vectors—the only two classes of delivery vehicles used for CAR transgene transfer into T cells in all approved CAR-T products [4]. Thus, developing cost-effective and scalable vector production methods is critically important for expanding access to this life-saving technology and reducing its cost.

LVs engineered from the HIV-1 genome remain the “gold standard” for genetic modification of T cells. Their key advantages include the ability to efficiently transduce both dividing and non-dividing cells, as well as stable, long-term transgene expression through host genome integration [5,6]. These properties make LVs indispensable for generating CAR-T products that require sustained CAR expression throughout cell generations. Moreover, recent studies demonstrate the expansion of CAR-T therapy beyond oncology—into autoimmune diseases (systemic lupus erythematosus, rheumatoid arthritis)—creating additional demand for clinical-grade LVs [3].

In academic laboratories, LV production is most commonly achieved via plasmid transfection of adherent HEK293 cells and their derivatives cultured in static vessels [7,8]. However, this approach suffers from fundamental drawbacks that render it highly inefficient for industrial scaling. First, adherent monolayers are difficult to scale beyond 10^9^ cells, necessitating multi-tiered plastic culture vessels (e.g., Cell Factory systems). This increases labor costs, consumable consumption, and production time. Second, the dependence of adherent cells on fetal bovine serum (FBS) creates risks of adventitious virus contamination, causes batch-to-batch variability, and substantially increases costs [9,10]. Third, industrial-scale transfection of adherent cultures requires large quantities of high-quality plasmid DNA, imposing an additional burden on downstream purification steps and increasing overall cost.

To address these challenges, researchers are actively pursuing alternative LV production strategies suitable for large-scale cGMP manufacturing [11,12]. A natural and highly promising solution is the transition to suspension cultures, which are significantly easier to scale, enable the use of serum-free media (SFM), and integrate better with modern bioreactor systems. Many strategies employ HEK293 cells (or their derivatives) adapted to suspension growth [10,13,14]. Although suspension adaptation and transfection protocols for HEK cells exist, many of these protocols were designed for recombinant protein production, not for generating packaged LVs [7,8,15,16]. Moreover, adapting HEK293 cells to serum-free suspension conditions has been shown to reduce transfection efficiency [17,18,19].

Commercial suspension LV production systems (e.g., LV-MAX from Thermo Fisher) exist, but they require proprietary reagents, specialized media, and costly licensing agreements, placing them out of reach for most academic institutions and resource-limited laboratories [20]. Consequently, there is an urgent need to develop an open, accessible, and scalable LV production platform based on suspension-adapted HEK293 cells—one that combines high efficiency with low cost and can be readily reproduced in academic settings.

The aim of this study was to adapt HEK293FT cells to suspension growth in serum-free medium (FreeStyle 293 Expression Medium) and to employ the adapted cells for LV production carrying a CAR transgene for CAR-T therapy. Using a direct adaptation method, we generated a novel cell line, HEK293FT-DS, that grows stably in suspension with high viability. We thoroughly characterized the growth parameters and stability of the resulting cell lines, optimized key transfection parameters (PEI:DNA ratio, cell density at transfection), and successfully scaled the process to obtain LV quantities sufficient for transducing primary T cells on the CliniMACS Prodigy instrument. The functional potency of the resulting vectors was confirmed by their ability to efficiently transduce donor T cells, achieving CAR expression levels > 57%. Our HEK293FT-DS-based platform represents a cost-effective and accessible alternative to commercial systems, positioning it as a valuable tool for academic CAR-T cell production.

## 2. Materials and Methods

### 2.1. Cell Lines and Reagents

Adherent HEK293FT cells (Thermo Fisher Scientific Cat. R70007, Waltham, MA, USA) were cultured in complete medium, which is Dulbecco’s Modified Eagle’s Medium (DMEM) with high glucose (Gibco, Grand Island, NY, USA, Cat. 11965092), supplemented with 10% fetal bovine serum (FBS; Gibco Cat. 10091148, Grand Island, NY, USA), 1% penicillin–streptomycin (Pen/Strep), 1% non-essential amino acids (NEAA), and 2 mM L-glutamine (all from Gibco, Grand Island, NY, USA). Cells were maintained at 37 °C in a 5% CO_2_ atmosphere.

Suspension-adapted cell lines derived from HEK293FT by the methods of sequential and direct adaptation were cultured in serum-free FreeStyle 293 Expression Medium (SFM; Gibco, Grand Island, NY, USA, Cat. 12338026). Cells were grown in 250 mL Erlenmeyer flasks with vented caps (Corning, Corning, NY, USA, Cat. 431144) using a working culture volume of 50 mL. The flasks were placed on an orbital shaker (IKA, Staufen im Breisgau, Germany, model KS260CS1) installed in a CO_2_ incubator (Wiggens, Wuppertal, Germany, model WCI-1200). Cultures were agitated constantly at 150 rpm. Cells were split when the density reached 2 × 10^6^ cells/mL; the next passage was performed at an initial seeding density of 3 × 10^5^ cells/mL. Cell viability was regularly determined by trypan blue (Sigma-Aldrich, St. Louis, MO, USA) exclusion.

### 2.2. Plasmids

The following plasmids were used: the LV-RNA-encoding transfer vectors LV/CAR and LV/CAR-GFP were described in a previous article [21]. Both vectors encode a chimeric antigen receptor (CAR) against the human cell surface marker CD19 as the transgene. LV/CAR-GFP additionally encodes GFP fused to CAR via an uncleavable linker [21]. For LV packaging, the packaging helper psPAX2 (Addgene, Watertown, MA, USA, Cat. 12260) and the envelope plasmid pMD2.G (Addgene, Watertown, MA, USA, Cat. 12259) were used. Plasmids were isolated by alkaline lysis and purified by ultracentrifugation in cesium chloride gradients as described in [22].

### 2.3. Sequential Adaptation of HEK293FT Cells to Suspension Culture

Adherent HEK293FT cells were grown in five 150 mm dishes in complete DMEM until approximately 90% confluence. The cells were detached using TrypLE Select (Gibco, Grand Island, NY, USA, Cat. 12563-029), pooled, counted, and transferred into a 500 mL Erlenmeyer shaker flask (Corning, Corning, NY, USA, Cat. 431145) containing 100 mL of complete DMEM. The seeding density was 1 × 10^6^ cells/mL. The flask was placed on an orbital shaker installed in a CO_2_ incubator and incubated at 150 rpm and 37 °C in 5% CO_2_.

Every 3–6 days, or when the cell density exceeded 2 × 10^6^ cells/mL, the culture was passaged. During seven passages, the proportion of serum-containing medium was gradually reduced while the proportion of serum-free FreeStyle 293 Expression Medium (SFM) was increased: P1—0% SFM; P2—5% SFM; P3—10% SFM; P4—25% SFM; P5—50% SFM; P6—75% SFM; P7—100% SFM. Viable cell density and viability were monitored before and at the end of each passage by trypan blue exclusion. After P7, cells were cryopreserved in SFM supplemented with 10% human serum albumin (HSA; Biopharma Plasma LLC, Bila Tserkva, Ukraine) and 10% DMSO (Sigma-Aldrich, St. Louis, MO, USA).

### 2.4. Direct Adaptation of HEK293FT Cells to Suspension Culture

HEK293FT cells were grown in five 150 mm dishes in complete DMEM to approximately 90% confluence, detached using TrypLE Select, pooled and transferred into a 500 mL Erlenmeyer shaker flask containing 100 mL of FreeStyle 293 Expression Medium (SFM). The flask was placed on an orbital shaker at 150 rpm and incubated at 37 °C in 5% CO_2_.

Passaging was performed every four days for passages P1–P6, and the final passage (before cryopreservation) lasted 5 days. Before each passage, the viable cell density was determined. To initiate a new passage, 50 mL of the culture was transferred to a new flask and supplemented with 50 mL of fresh SFM. After P7, cells were cryopreserved in SFM supplemented with 10% HSA and 10% DMSO.

### 2.5. Comparative Growth Kinetics of Adapted Cell Lines

Cell lines obtained using the sequential adaptation method (designated HEK293FT-SS) and the direct adaptation method (designated HEK293FT-DS) were subjected to batch growth analysis. Cryopreserved vials were thawed and cultured in SFM for two 3-day passages to allow recovery.

For growth kinetics, cells were seeded into 250 mL Erlenmeyer flasks (Corning Cat. 431144) containing 50 mL of SFM at an initial density of 3 × 10^5^ cells/mL. Flasks were incubated at 150 rpm, 37 °C, and 5% CO_2_. Every 24 h for 10 days, aliquots were taken to measure viable cell density and viability using trypan blue exclusion. Three biological replicates were performed.

### 2.6. Stability of Growth Characteristics over 35 Passages

This experiment was performed with HEK293FT-DS cells. Cells were seeded into 250 mL Erlenmeyer flasks containing 50 mL of SFM at 3 × 10^5^ cells/mL and incubated for 3 days. At the end of each passage, the viable cell density was measured, and the cells were reseeded for the next passage at 3 × 10^5^ cells/mL.

The specific growth rate μ (day^−1^) was calculated as:

μ = ln(N/300,000)/3

The population doubling time Td (days) was calculated as:

Td = ln(2)/μ × 24

### 2.7. Preparation of PEI Transfection Reagent

All transfections were carried out using linear 40 kDa polyethylenimine (PEI MAX; Polysciences Cat. 24765-1; Warrington, PA, USA). The PEI stock solution (1 mg/mL) was prepared in 25 mM HEPES (Sigma-Aldrich, St. Louis, MO, USA, pH 7.5) containing 150 mM NaCl (Sigma-Aldrich, St. Louis, MO, USA), sterilized by filtration (0.22 μm), and stored at +4 °C.

### 2.8. Transfection of Adherent HEK293FT Cells

Adherent HEK293FT cells were seeded in 150 mm dishes (P150) at a density of 1 × 10^5^ cells/cm^2^.

For each P150 dish, a total of 10 μg of plasmid DNA (pLV/CAR-GFP, psPAX2, and pMD2.G at a molar ratio of 2:1.5:1) was diluted in 2.5 mL of serum-free DMEM. Separately, 30 μL of the 1 mg/mL PEI MAX stock solution was diluted in 2.5 mL of serum-free DMEM. The diluted PEI solution was then added to the diluted DNA solution and mixed briefly by vortexing. The resulting mixture was incubated for 15 min at room temperature (RT) to allow polyplex formation.

Before addition of the transfection mixture, the culture medium was removed. Immediately, 5 mL of the polyplex mixture was added to the cell monolayer and distributed evenly over the cells. The cells were incubated for 4 h at 37 °C in a 5% CO_2_ atmosphere. Subsequently, 20 mL of complete DMEM was added to each dish without removing the transfection mixture. Cells were further incubated for 48 h. The culture supernatant was then collected, clarified by centrifugation at 300× *g* for 10 min, filtered through a 0.45 μm PES membrane (Pall Corporation Cat. 515-0157, Port Washington, NY, USA), aliquoted, and stored at −80 °C until use. All transfections were performed in three biological replicates (independent transfections with separate cell passages).

### 2.9. Initial Transfection Protocol for Suspension-Adapted Cells

Suspension-adapted cells were transfected using PEI MAX. Cells were routinely maintained in SFM and passaged every 2–3 days to maintain viability above 90%.

On the day of transfection, cells were seeded into 250 mL Erlenmeyer flasks at a density of 1 × 10^6^ viable cells/mL in a final volume of 50 mL of fresh SFM. A plasmid mixture containing the transfer vector pLV/CAR-GFP, the packaging helper psPAX2, and the envelope plasmid pMD2.G at a molar ratio of 2:1.5:1 was prepared. The total amount of plasmid DNA was 1 μg per 10^6^ cells. This DNA mixture was diluted in 5 mL of SFM. Separately, PEI MAX (3 μg per 10^6^ cells) was diluted in 5 mL of SFM. The DNA and PEI solutions were combined, vortexed and incubated for 15 min at RT. The polyplex mixture was then added to the cells. After 48 h of incubation at 37 °C with agitation at 150 rpm, the culture supernatant was harvested, clarified by centrifugation at 300× *g* for 10 min, filtered through a 0.45 μm PES membrane, aliquoted, and stored at −80 °C. Transfections were performed in three biological replicates.

### 2.10. Optimization of Transfection Conditions

Optimization of the PEI:DNA mass ratio. For adherent cells, the protocol described in Section 2.8 was used. For suspension cells, the initial protocol described in Section 2.9 served as the starting point. Different PEI:DNA mass ratios (1:1, 1.5:1, 2:1, 2.5:1, 3:1, and 4:1) were tested. The total amount of plasmid DNA (pLV/CAR-GFP, psPAX2, and pMD2.G at a 2:1.5:1 molar ratio) was kept constant at 1 μg per 10^6^ cells, while the amount of PEI MAX was adjusted accordingly. All other parameters remained unchanged.

Optimization of cell density during transfection. Suspension-adapted cells were maintained in Erlenmeyer flasks in SFM with viability consistently above 90%. On the day of transfection, the required number of cells was pelleted by centrifugation (300× *g*, 5 min) and resuspended in either fresh SFM or complete DMEM to a volume of 40 mL, yielding cell densities ranging from 0.625 × 10^6^ to 30 × 10^6^ cells/mL. The amounts of PEI and total plasmid DNA were scaled proportionally to the cell density, maintaining a constant DNA amount of 1 μg per 10^6^ cells and a constant PEI:DNA mass ratio of 2.5:1. Polyplexes were formed by mixing 5 mL of the DNA solution in SFM with 5 mL of the PEI solution in SFM, followed by incubation for 15 min at RT. The resulting 10 mL of polyplex mixture was added to the 40 mL of cell suspension, giving final working cell densities of 0.5 × 10^6^ to 24 × 10^6^ cells/mL. Four hours after addition of the polyplex mixture, the culture was diluted with SFM to a final density of 1 × 10^6^ cells/mL. The LV-containing supernatant was harvested at 48 h post-transfection and processed as described in Section 2.8. Each condition was tested in three biological replicates.

### 2.11. Scale-Up of Lentiviral Vector Production

For large-scale production of the LV/CAR vector, HEK293FT-DS cells were maintained in 1 L Erlenmeyer flasks (Corning Cat. 431146) with a working culture volume of 250 mL. On the day of transfection, 250 × 10^6^ cells per sample were pelleted by centrifugation (300× *g*, 5 min) and resuspended in 14 mL of SFM, corresponding to a cell density of 17.9 × 10^6^ cells/mL. The cell suspension was transferred to a 125 mL Erlenmeyer flask (Corning Cat. 431143) and placed on an orbital shaker. The DNA (250 μg) and PEI MAX (625 μg) were each diluted in 800 μL of SFM. The two solutions were combined and incubated for 15 min at RT, and the resulting polyplex mixture was added to the cells, yielding a final cell density of 16 × 10^6^ cells/mL. Four hours after addition of the polyplex mixture, the culture was transferred to a 1 L Erlenmeyer flask, and fresh SFM was added to bring the final volume to 250 mL.

Cultures were harvested 48 h post-transfection. Four identical samples were processed simultaneously to collect a total of 1 L of LV-containing supernatant.

### 2.12. Concentration of Lentiviral Vectors

Clarified supernatant containing lentiviral particles was concentrated using a two-step procedure: first by tangential flow filtration (TFF), followed by ultracentrifugation at 20,000× *g*.

For TFF, the supernatant was processed on a Tanfil 100 tangential flow filtration system (Rocker Scientific Co., New Taipei City, Taiwan) equipped with a Minimate TFF capsule containing an Omega membrane (Pall Life Sciences Cat. OA300C12, 300 kDa molecular weight cut-off). A 500 mL aliquot of clarified supernatant was concentrated to a final volume of 7 mL (the system dead volume). The reservoir was then rinsed with 50 mL of phosphate-buffered saline (PBS; Gibco, Grand Island, NY, USA) supplemented with 1% human serum albumin (HSA), and the concentration procedure was repeated. The resulting 7 mL concentrate was collected and pooled with the previous concentrate. The remaining supernatant was processed similarly. In total, 1 L of supernatant was concentrated to 28 mL (concentration factor 35.7×).

The TFF concentrate was transferred in 1 mL aliquots into thick-walled 1.5 mL centrifuge tubes (Beckman Coulter, Brea, CA, USA, Cat. 357448) and subjected to ultracentrifugation at 20,000× *g* for 2 h at 4 °C using a Beckman MLA-130 rotor (Beckman Coulter, Brea, CA, USA) and Optima MAX-XP Ultracentrifuge (Beckman Coulter, Brea, CA, USA). After centrifugation, the supernatant was removed. The resulting pellets were resuspended overnight in 0.5 mL of RPMI 1640 medium (Gibco, Grand Island, NY, USA) supplemented with 10% HSA and 0.0001% Pluronic F68 (Sigma-Aldrich, St. Louis, MO, USA, Cat. P5556). The next day, the resuspended LV particles were pooled, aliquoted and stored at −80 °C.

### 2.13. Determination of Lentiviral Functional Titer

Lentiviral functional titers (TU/mL) were determined by transducing HEK293FT cells with serial dilutions, followed by flow cytometric analysis. HEK293FT cells were seeded in 6-well plates at 4 × 10^5^ cells per well and allowed to attach. Serial dilutions of LV preparations were prepared in complete DMEM containing 8 µg/mL polybrene (Sigma-Aldrich, St. Louis, MO, USA, Cat. H9268) and added to the cells. After 12 h, the polybrene-containing medium was replaced with standard complete DMEM. Preliminary experiments used dilutions from 1:10 to 1:10,000 to estimate the titer range. Subsequently, titrations were performed with dilutions of 1:10 to 1:1000 to achieve 1–20% transduced cells in the final well. At 72 h post-transduction, cells were harvested.

For the LV/CAR-GFP vector, transduction efficiency was assessed by quantifying GFP-positive cells. Cells were detached with trypsin-EDTA (Gibco, Grand Island, NY, USA), washed with PBS, and resuspended in MACSQuant Running Buffer (RB; Miltenyi Biotec, Bergisch Gladbach, Germany, Cat. 130-092-747). For the LV/CAR vector (without GFP), non-permeabilized cells were stained sequentially with biotinylated CD19 CAR Detection Reagent (Miltenyi Biotec, Bergisch Gladbach, Germany, Cat. 130-129-550) and anti-biotin-PE antibody (Miltenyi Biotec, Bergisch Gladbach, Germany, Cat. 130-113-291). Samples were acquired on a MACSQuant 10 flow cytometer (Miltenyi Biotec, Bergisch Gladbach, Germany). At least 50,000 events were recorded per sample. Data were analyzed using MACSQuantify Software v.2.13 (Miltenyi Biotec, Bergisch Gladbach, Germany). Gates for positive cells were set using naïve HEK293FT cells as a negative control, with <0.1% events in the positive gate. MACSQuant Calibration Beads (Miltenyi Biotec, Bergisch Gladbach, Germany, Cat. 130-093-607) were used to monitor gate stability across experiments. The functional titer (TU/mL) was calculated as:

TU/mL = (% positive cells/100) × (number of cells at transduction) × (dilution factor)

### 2.14. p24 ELISA and Calculation of Specific Activity

The concentration of HIV-1 p24 capsid protein in LV batches was measured using a Lenti-X p24 Rapid Titer Kit (Takara Bio USA, Mountain View, CA, USA, Cat. 631476) according to the manufacturer’s instructions. Briefly, serial 10-fold dilutions of the samples were prepared in the diluent buffer provided with the kit. Aliquots (100 µL) of diluted samples were added to anti-p24-coated wells of the ELISA plate, followed by 20 µL of lysis buffer, and incubated for 30 min. Then, 100 µL of HRP-conjugated anti-p24 antibody was added and incubated for an additional 30 min. After six washes with wash buffer, 100 µL of 3,3′,5,5′-tetramethylbenzidine (TMB) substrate was added, and incubated for 30 min in the dark, and the reaction was stopped with 100 µL of stop solution. Optical density was measured at 450 nm. p24 concentration (ng/mL) was calculated using a standard curve (0–1600 pg/mL). Specific activity was defined as the ratio of functional titer to p24 concentration in the same sample:

Specific activity (TU/pg p24) = Functional titer (TU/mL)/p24 concentration (pg/mL)

### 2.15. Functional Characterization of the LV/CAR Vector

Primary human T-cells for CAR-T cell generation were isolated from concentrated peripheral blood leukocytes (an apheresis product) provided by the Scientific and Production Center of Transfusiology (SPCT, Astana, Kazakhstan), a licensed medical institution. Donors provided written informed consent at the time of collection. Only surplus, anonymized, frozen apheresis products not required for clinical use were transferred to the authors’ laboratory for this work. No blood products were collected specifically for this study. The study protocol was approved by the Institutional Ethics Committee of the National Center for Biotechnology (NCB).

Isolation of CD4^+^ and CD8^+^ T cells was performed on a CliniMACS Prodigy cell processor (Miltenyi Biotec, Bergisch Gladbach, Germany) using a TS520 Tubing Set (Miltenyi Biotec, Bergisch Gladbach, Germany) according to the manufacturer’s instructions, as described in detail in [23]. Briefly, a cryopreserved leukapheresis product was thawed, treated with Benzonase (Merck KGaA, Darmstadt, Germany, 50 U/mL; Cat. 70664-3), and washed. CD4^+^ and CD8^+^ T cells were isolated immunomagnetically using the automated T-Cell Transduction 2.0 (TCT 2.0) program built into the CliniMACS Prodigy instrument. The isolated T cells were transferred to a reapplication bag (RAB) connected to the TS520 tubing set. The T cell suspension in TexMACS Medium (Miltenyi Biotec, Bergisch Gladbach, Germany, Cat. 130-097-196) was supplemented with 10% HSA and 10% DMSO, aliquoted, and cryopreserved in the vapor phase of liquid nitrogen.

For CAR-T cell production, previously isolated CD4^+^ and CD8^+^ T cells were recovered from cryostorage. A total of 1 × 10^6^ T cells were transferred into TexMACS Medium supplemented with 1000–1500 U/mL recombinant human IL-7 (Miltenyi Biotec, Bergisch Gladbach, Germany, Cat. 170-076-111) and 300–450 U/mL recombinant human IL-15 (Miltenyi Biotec, Bergisch Gladbach, Germany, Cat. 170-076-186). The cells were activated with TransAct reagent (Miltenyi Biotec, Bergisch Gladbach, Germany, Cat. 130-128-758). On the next day, cells were transduced with the LV/CAR vector prepared as described in Section 2.11 and Section 2.12. A total of 2 × 10^6^ transducing units (TU) of the vector were used to achieve a multiplicity of infection (MOI) of 2 TU/cell. The culture was expanded in T-flasks until day 12, and the final CAR-T cell product was cryopreserved in TexMACS Medium containing 10% HSA and 10% DMSO.

### 2.16. Flow Cytometric Analysis of CAR Expression

The final CAR-T cell product was analyzed by flow cytometry as described in [23]. Cells were stained with the following antibodies (all from Miltenyi Biotec, Bergisch Gladbach, Germany): CD3-FITC (130-113-138), CD4-VioGreen (130-113-221), CD8-APC-Vio770 (130-113-155), CD14-APC (130-113-143), CD45-VioBlue (130-113-122), biotinylated CD19 CAR Detection Reagent (130-129-550), and anti-biotin-PE (130-113-291). CAR staining was performed in two steps: first with biotinylated CD19 CAR Detection Reagent (2 µL per 100 µL cell suspension, 10 min at RT), followed by washing and incubation with anti-biotin-PE together with the remaining antibodies (10 min at RT). After a final wash, cells were resuspended in MACSQuant Running Buffer (Miltenyi Biotec, Bergisch Gladbach, Germany, Cat. 130-092-747). Samples were acquired on a MACSQuant 10 flow cytometer (Miltenyi Biotec, Bergisch Gladbach, Germany), and data were analyzed using MACSQuantify Software v.2.13 (Miltenyi Biotec, Bergisch Gladbach, Germany).

### 2.17. Statistical Analysis

Data from three biological replicates are presented as mean ± SD. Comparisons between two groups were made using an unpaired two-tailed Student’s *t*-test. For multiple groups, one-way ANOVA with Tukey’s post-hoc test was used. Two-way ANOVA was used to analyze the transfection optimization data (factors: cell density and culture medium). For growth kinetics over time, two-way repeated-measures ANOVA (RM ANOVA) was performed with factors “cell line” and “time”, applying the Geisser–Greenhouse correction. One-way RM ANOVA with the Geisser–Greenhouse correction was applied to stability parameters over 15 passages. All statistical analyses were performed using GraphPad Prism 9.3.1 (GraphPad Software, San Diego, CA, USA). Significance levels are designated as: *ns* (not significant, *p* > 0.05), * (*p* ≤ 0.05), ** (*p* ≤ 0.01), *** (*p* ≤ 0.001).

## 3. Results

### 3.1. Sequential Adaptation of HEK293FT Cells to Serum-Free Suspension Culture

**Figure 1:**
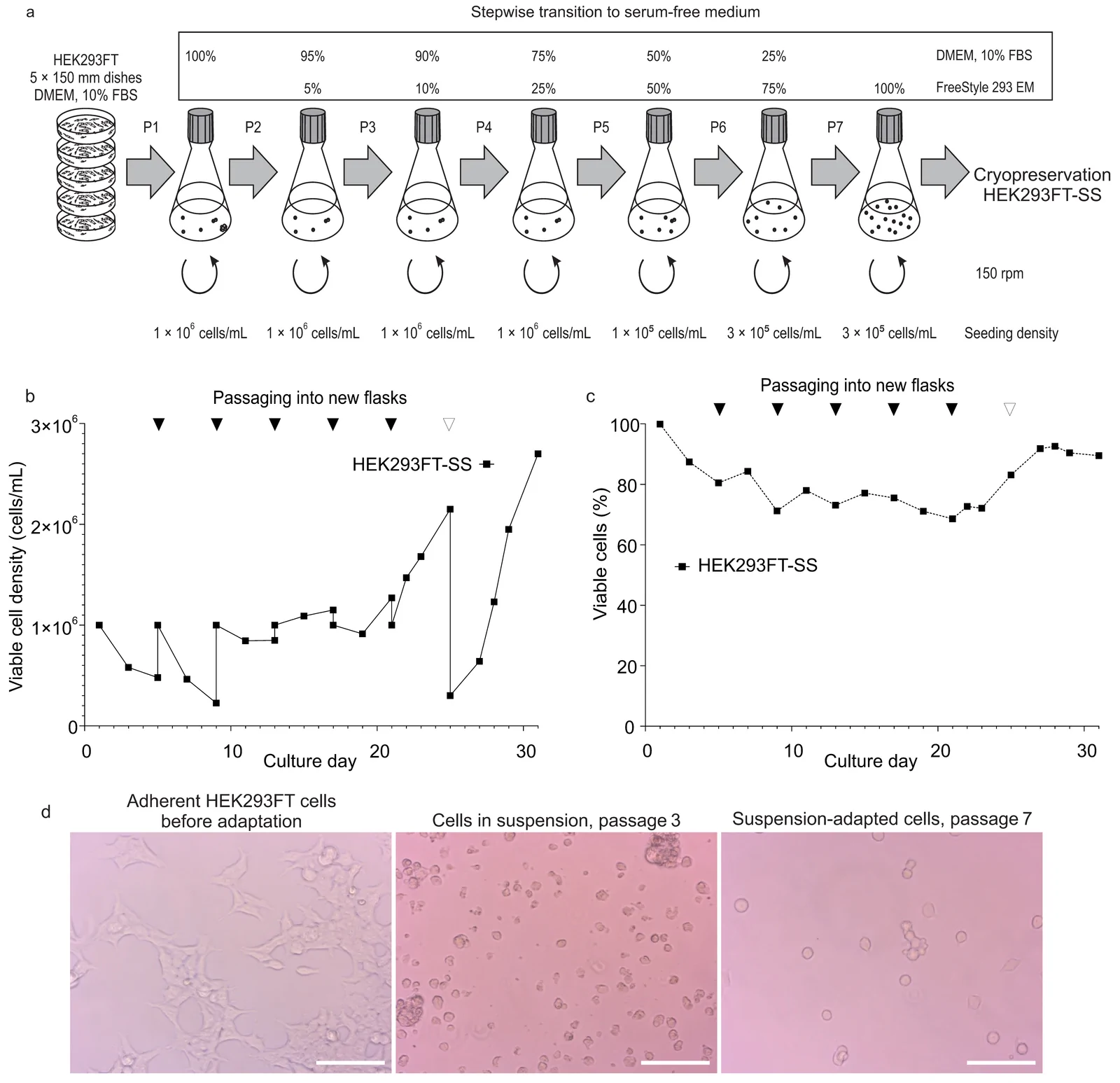
Sequential adaptation of HEK293FT cells to serum-free suspension culture. (**a**) Schematic of the adaptation protocol. Starting from an adherent culture (~1 × 10^8^ cells), cells were passaged seven times with a stepwise increase in serum-free FreeStyle 293 Expression Medium (SFM) from 0% to 100% (exact percentages are indicated). Passaging was performed every 3–6 days, or when the cell density exceeded 2 × 10^6^ cells/mL. Initial seeding densities for each passage are shown below the curved arrows. (**b**,**c**) Adaptation was performed once; data points represent individual measurements. (**b**) Viable cell density over time. Cells were seeded at 1 × 10^6^ cells/mL on day 1. Passages (P1–P7) are indicated; triangles mark the days on which passages were initiated. Black triangles: reseeding at 1 × 10^6^ cells/mL; white triangles: reseeding at 3 × 10^5^ cells/mL. (**c**) Cell viability (%) corresponding to the time course shown in (**b**). (**d**) Morphology of HEK293FT cells before, during and after adaptation. Phase-contrast images (20× objective, scale bar = 100 μm). Left: parental adherent cells. Center: cells at passage 3 (early adaptation). Right: suspension-adapted cells at passage 7 (designated HEK293FT-SS). Adapted cells grow as single cells or loose clusters with high viability.

To adapt HEK293FT cells to serum-free suspension growth, we employed a stepwise reduction in serum-containing DMEM while gradually increasing the proportion of serum-free FreeStyle 293 Expression Medium (SFM; Gibco, Grand Island, NY, USA) from 0% to 100% (P1: 0% SFM; P2: 5%; P3: 10%; P4: 25%; P5: 50%; P6: 75%; P7: 100%, Figure 1a). The resulting viable cell density and cell viability are shown in Figure 1b,c. The culture initially suffered a sharp decline in both parameters but gradually recovered and entered exponential growth, reaching a maximum density of 2.7 × 10^6^ cells/mL with 89.5% viability by day 31.

At the early stages of adaptation, large cell clumps were observed; however, as passaging progressed, the size of cell aggregates gradually decreased (Figure 1d). By the end of the 7th passage, the suspension-adapted cell line, designated HEK293FT-SS, consisted mainly of single cells with occasional small aggregates (Figure 1d, right panel). Cells within the aggregates were loosely associated, as they readily dissociated upon gentle pipetting.

### 3.2. Direct Adaptation of HEK293FT Cells to Suspension Culture

**Figure 2:**
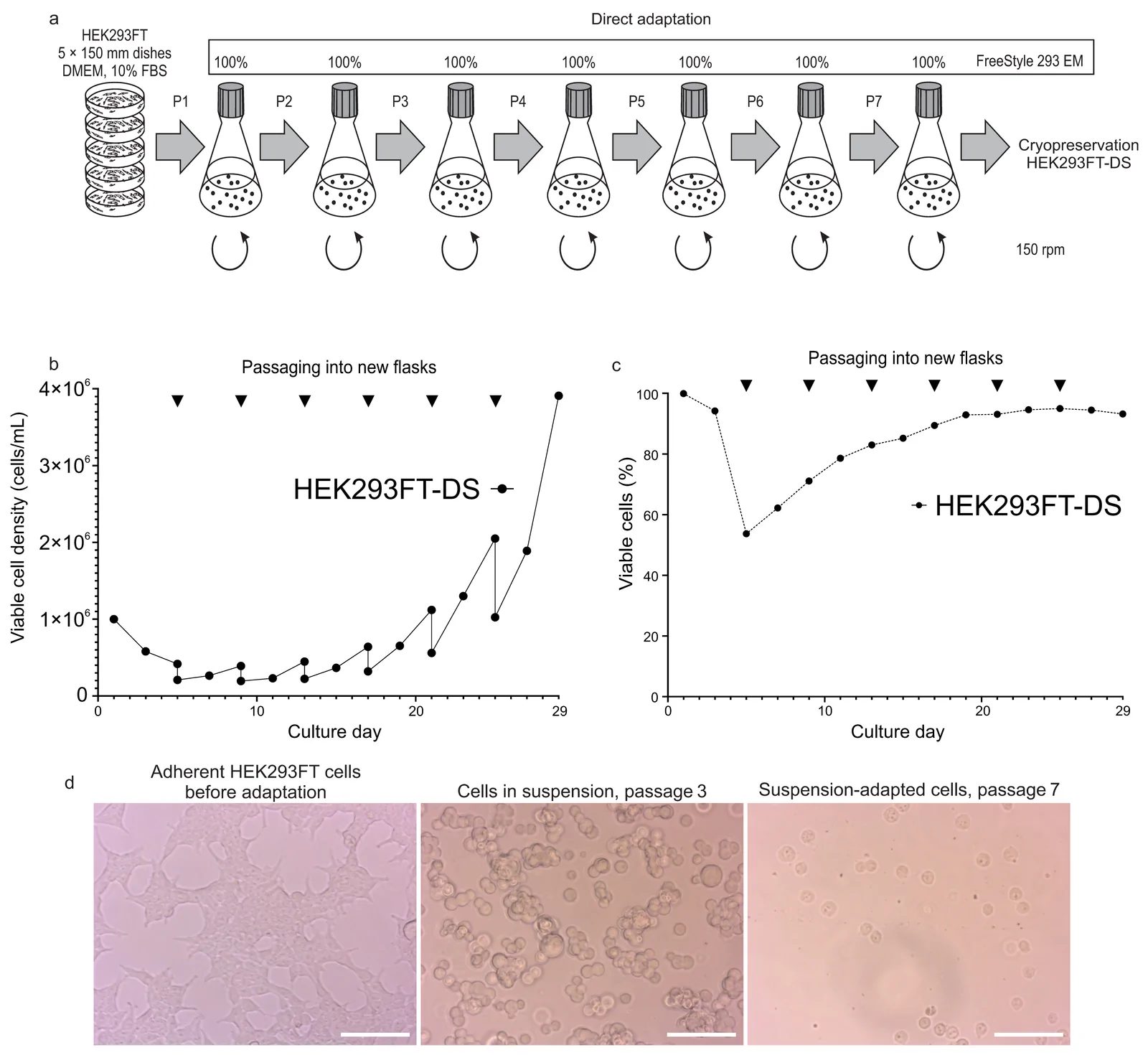
Direct adaptation of HEK293FT cells to serum-free suspension culture. (**a**) Schematic of the adaptation protocol. Starting from an adherent culture (~1 × 10^8^ cells), cells were detached and transferred directly into 100% serum-free FreeStyle 293 Expression Medium (SFM) and passaged every 4 days by dilution with fresh SFM (transfer of half the culture volume into an equal volume of fresh SFM). (**b**,**c**) Adaptation was performed once; data points represent individual measurements. (**b**) Viable cell density over time. Cells were seeded at 1 × 10^6^ cells/mL on day 1. Passages (P1–P7) are indicated; triangles mark the days on which passages were initiated. (**c**) Cell viability (%) corresponding to the time course shown in (**b**). (**d**) Morphology of HEK293FT cells before, during and after adaptation. Phase-contrast images (20× objective, scale bar = 100 μm). Left: parental adherent cells. Center: cells at passage 3 (early adaptation). Right: fully adapted cells at passage 7 (designated HEK293FT-DS). Adapted cells grow predominantly as single cells with some loose clusters and exhibit high viability.

In parallel with sequential adaptation, we tested a direct adaptation strategy: adherent cells were detached and transferred directly into 100% SFM, as illustrated in Figure 2a. The initial seeding density was the same as in the sequential protocol (1 × 10^6^ cells/mL). Passaging was performed every 4 days by transferring half of the culture volume into an equal volume of fresh SFM. Viable cell density and viability during this process are shown in Figure 2b,c.

Following transfer into SFM, the culture exhibited an immediate stress response: viable cell density and viability dropped sharply (Figure 2c). Nevertheless, the cells gradually recovered and, from the beginning of the third week, entered a robust growth phase. By the end of the observation period, the culture reached a maximum density of 3.91 × 10^6^ cells/mL with 93.2% viability. By passage 7, the culture had converted to a homogeneous suspension of predominantly single cells with occasional small clusters (Figure 2d). The directly adapted cell line was designated HEK293FT-DS and cryopreserved for further experiments.

Both adaptation protocols successfully generated suspension-adapted HEK293FT cells. However, direct adaptation yielded a fully adapted culture more rapidly. Moreover, the directly adapted cells at passage 7 showed less aggregation and a higher final viability compared to the sequentially adapted cells.

### 3.3. Growth Characteristics and Stability of Adapted Cell Lines

**Figure 3:**
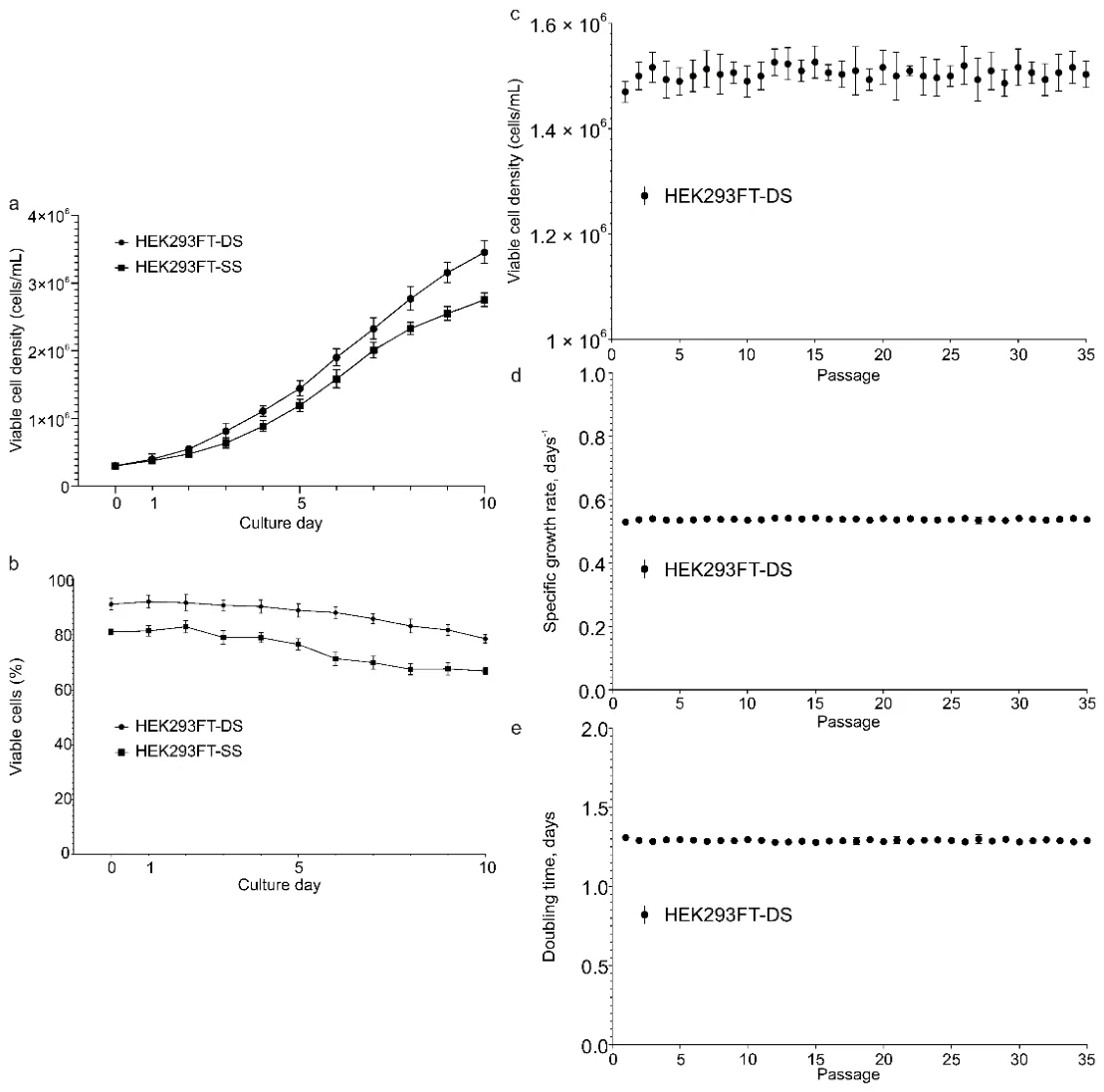
Growth kinetics of suspension-adapted HEK293FT-SS and HEK293FT-DS cells. Cells were seeded at 3 × 10^5^ cells/mL and monitored for 11 days without medium replacement. (**a**) Viable cell density (cells/mL). (**b**) Cell viability (%). Data are mean ± SD from three biological replicates (*n* = 3). For panels (**a**,**b**), a two-way repeated-measures ANOVA revealed significant main effects of cell line (SS vs. DS; *p* < 0.001) and time (*p* < 0.001), as well as a significant interaction (*p* < 0.001). (**c**–**e**) Stability of growth properties of HEK293FT-DS cells over 35 consecutive passages. Cells were passaged every 3 days at an initial density of 3 × 10^5^ cells/mL. (**c**) Viable cell density at the end of each passage, (**d**) specific growth rate μ (day^−1^), and (**e**) population doubling time Td (days). Data are mean ± SD (*n* = 3). HEK293FT-DS cells exhibited consistent growth parameters over 35 passages, indicating phenotypic stability. For panels (**c**–**e**), a one-way repeated-measures ANOVA (RM ANOVA) revealed no effect of passage number on viable cell density, specific growth rate, or doubling time.

To compare growth performance, HEK293FT-SS and HEK293FT-DS cells were seeded at 3 × 10^5^ cells/mL and cultured for 10 days without medium replacement (Figure 3a,b). Both cell lines exhibited typical sigmoidal growth curves. HEK293FT-DS cells reached a significantly higher maximum viable cell density than HEK293FT-SS cells (3.45 ± 0.07 vs. 2.75 ± 0.04 × 10^6^ cells/mL on day 10, *p* < 0.001). Viability remained above 90% for HEK293FT-DS cells throughout the experiment, whereas HEK293FT-SS cells declined to 83–84% by day 10. Therefore, HEK293FT-DS was selected as the primary cell line for all subsequent experiments.

To assess phenotypic stability, HEK293FT-DS cells were cultured for 35 consecutive passages. Cells were passaged every 3 days at an initial density of 3 × 10^5^ cells/mL, and viable cell density, specific growth rate (μ), and population doubling time (Td) were recorded at the end of each passage (Figure 3c–e). Viable cell density remained consistent across passages, averaging (1.505 ± 0.028) × 10^6^ cells/mL at each passage end, with no significant effect of passage number (RM ANOVA, *p* = 0.648), attesting to phenotypic stability. The specific growth rate (μ) for HEK293FT-DS cells averaged 0.538 ± 0.006 day^−1^, and the doubling time (Td) was 30.95 ± 0.35 h. These results demonstrate that HEK293FT-DS cells exhibit robust adaptation and stable growth characteristics over extended culture (Table 1).

### 3.4. Optimization of Lentiviral Vector Production

**Figure 4:**
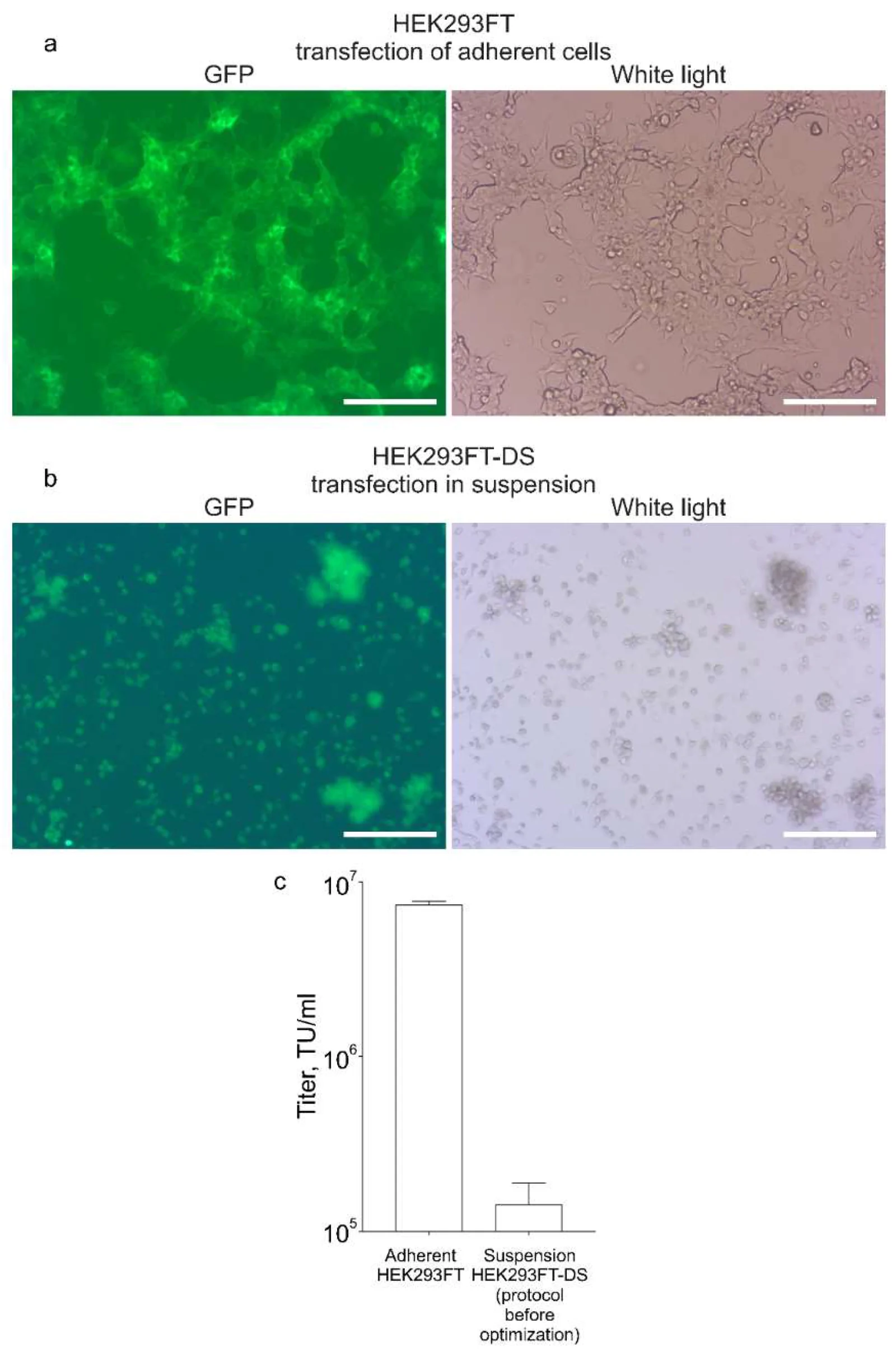
Lentiviral vector packaging efficiency in adherent HEK293FT cells and suspension-adapted HEK293FT-DS cells prior to optimization of transfection conditions. (**a**) Adherent HEK293FT cells were transfected with the transfer vector LV/CAR-GFP and packaging helpers using the protocol described in Section 2.8. Representative photographs (white light, left; GFP fluorescence, right) are shown. (**b**) Suspension-adapted HEK293FT-DS cells were transfected with the same LV packaging system using the protocol described in Section 2.9. Representative photographs (white light and GFP fluorescence) are shown. Images were acquired with a 20 × objective; scale bar = 100 μm. (**c**) Functional titers (TU/mL) of LV/CAR-GFP in culture supernatants. Data are mean ± SD from three biological replicates. Under these conditions, adherent cells produced substantially higher titers than suspension cells (*p* < 0.001).

We first compared LV titers produced by transfecting adherent HEK293FT cells (Section 2.8) and suspension-adapted HEK293FT-DS cells using the baseline protocol described in Section 2.9. As shown in Figure 4, adherent cells produced functional titers approximately 50-fold higher than suspension cells. These results clearly demonstrated the necessity of optimizing our transfection protocol for suspension cells to improve LV production.

**Figure 5:**
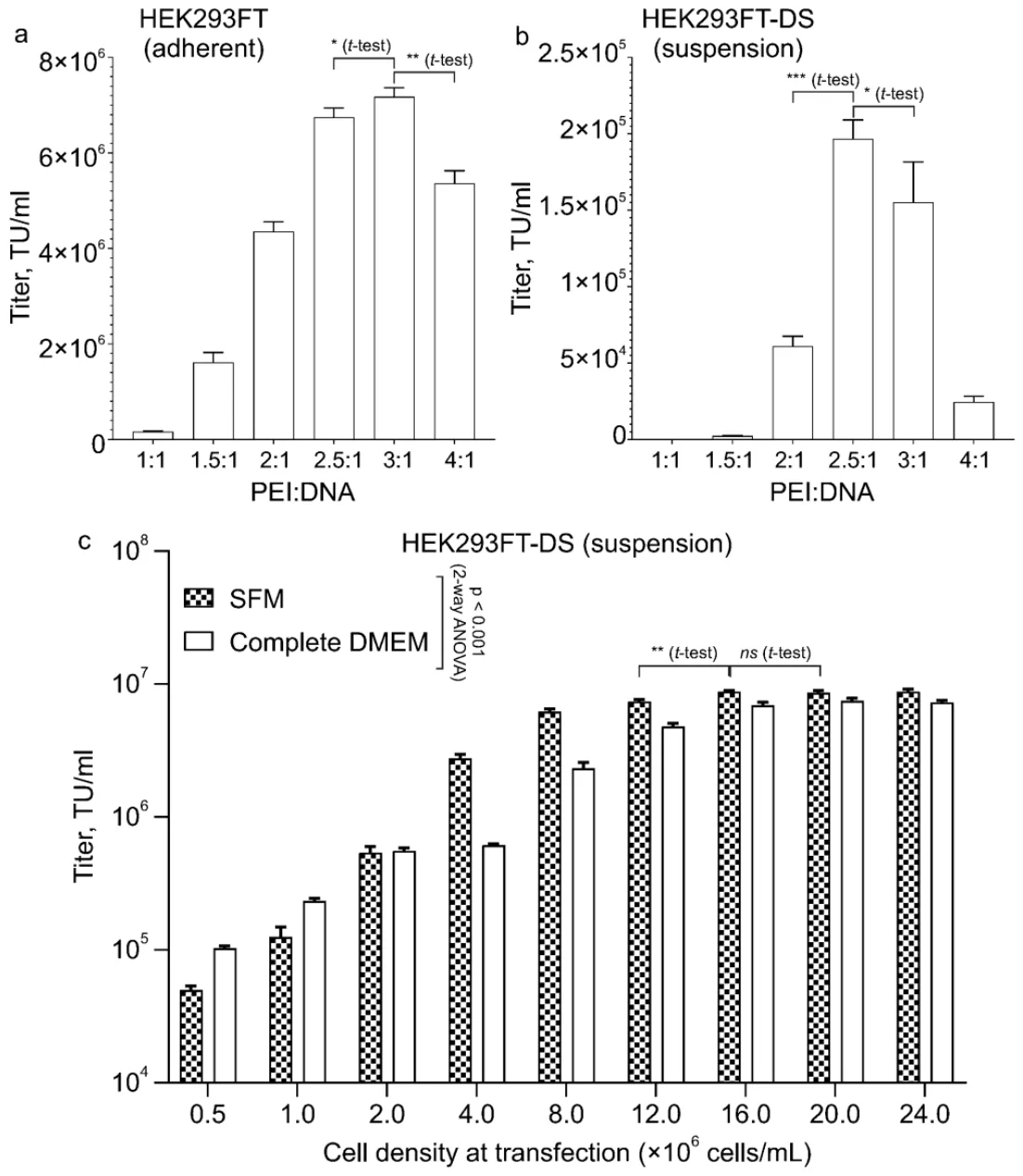
Optimization of transfection parameters for lentiviral vector production. (**a**,**b**) Effect of PEI:DNA mass ratio on functional titers in adherent HEK293FT cells (**a**) and suspension-adapted HEK293FT-DS cells (**b**). Supernatants were collected 48 h post-transfection and titered. Data are mean ± SD (*n* = 3). For suspension-adapted HEK293FT-DS cells, a PEI:DNA ratio of 2.5:1 yielded optimal titers (2.5:1 vs. 2:1, *p* < 0.001; 2.5:1 vs. 3:1, *p* = 0.038). (**c**) Effect of cell density at transfection on LV production in suspension-adapted cells. Transfections were performed in either SFM or complete DMEM, with constant DNA amount (1 μg/10^6^ cells) and PEI:DNA ratio (2.5:1). Cultures were diluted to 1 × 10^6^ cells/mL 4 h after polyplex addition. Supernatants were collected at 48 h and titered. Data are mean ± SD (*n* = 3). For panel (**c**), a two-way ANOVA (factors: cell density and culture medium) revealed significant main effects of cell density (*p* < 0.0001) and medium (*p* < 0.0001), as well as a significant interaction (*p* < 0.0001). Exploratory unpaired *t*-tests within the SFM condition revealed a significant difference between 12 × 10^6^ and 16 × 10^6^ cells/mL (*p* = 0.002), but no significant difference between 20 × 10^6^ and 16 × 10^6^ cells/mL (*p* = 0.508). Statistical significance is indicated as follows: ns (not significant, *p* > 0.05), * (*p* ≤ 0.05), ** (*p* ≤ 0.01), *** (*p* ≤ 0.001).

To determine the optimal PEI:DNA mass ratio, we transfected adherent and suspension-adapted cells at PEI:DNA ratios from 1:1 to 4:1 while keeping total DNA constant at 1 μg per 10^6^ cells (Figure 5a,b). The optimal ratio was 3:1 for adherent cells and 2.5:1 for suspension-adapted cells, the latter yielding functional titers of approximately 2.0 × 10^5^ TU/mL. For suspension-adapted cells, a ratio of 2.5:1 gave significantly higher titers than 2:1 (*p* < 0.001) and 3:1 (*p* = 0.038). Based on this result, all subsequent transfections of suspension cells used the ratio of 2.5:1.

We next examined the effect of cell density during transfection on functional LV titers in suspension-adapted cells using two media: SFM and complete DMEM. Cell density ranged from 0.5 × 10^6^ to 24 × 10^6^ cells/mL, while DNA amount (1 μg/10^6^ cells) and PEI:DNA ratio remained constant. Four hours after polyplex addition, cultures were diluted with SFM to 1 × 10^6^ cells/mL, and supernatants were collected after another 48 h.

Increasing cell density markedly improved LV titers in both media (Figure 5c). A two-way ANOVA (factors: cell density and culture medium) revealed significant main effects of cell density (*p* < 0.0001) and medium (*p* < 0.0001), as well as a significant interaction (*p* < 0.0001). In SFM, titers reached a plateau of approximately 9 × 10^6^ TU/mL at a density of 16 × 10^6^ cells/mL, with no significant increase at higher densities. Unpaired *t*-tests within the SFM condition showed a significant difference between 12 × 10^6^ and 16 × 10^6^ cells/mL (*p* = 0.002), but no significant difference between 20 × 10^6^ and 16 × 10^6^ cells/mL (*p* = 0.508). In DMEM, titers increased steadily to approximately 7 × 10^6^ TU/mL at 24 × 10^6^ cells/mL, though gains above 16 × 10^6^ cells/mL were marginal, and no clear saturation was observed. SFM consistently outperformed DMEM at high cell densities (>2 × 10^6^ cells/mL). Therefore, for large-scale LV packaging experiments, this density of 16 × 10^6^ cells/mL in SFM was used. Although complete DMEM also supported efficient transfection at high cell densities, SFM was selected for scale-up to maintain the serum-free advantage of the platform.

### 3.5. Scale-Up of Lentiviral Vector Production

Using the optimized transfection parameters, we scaled up LV/CAR production to obtain sufficient vector for clinical-scale CAR-T cell manufacturing on a CliniMACS Prodigy instrument. Two independent scale-up experiments were performed. In each experiment, four parallel transfections were carried out. For each transfection, 250 × 10^6^ HEK293FT-DS cells were transfected as described in Section 2.11, yielding 1 L of LV-containing supernatant per batch. The supernatant was clarified and concentrated using tangential flow filtration (TFF) followed by ultracentrifugation, as detailed in Section 2.12. The recovery at each processing step is summarized in Table 2.

Across the two experiments, the total functional yields after concentration were 4–5 × 10^9^ TU, corresponding to overall recoveries of 64–71%, relative to the starting supernatant. Thus, the optimized high-density transfection protocol and two-step concentration procedure consistently produce LV batches suitable for clinical-scale CAR-T cell production.

### 3.6. Comparison of Production Methods

To compare the specific activity of LVs produced in suspension cultures using the optimized protocol (Section 2.11) against the conventional adherent system (Section 2.8), we performed parallel LV production experiments. The functional titers obtained from the adherent culture supernatant were approximately 1.4-fold higher than those from the suspension culture (1.29 ± 0.21 × 10^7^ TU/mL vs. 9.0 ± 1.5 × 10^6^ TU/mL; *p* < 0.05). However, p24 ELISA revealed that the suspension culture supernatant contained significantly more p24 antigen (652 ± 89 ng/mL) compared to the adherent culture (318 ± 42 ng/mL; *p* < 0.01), reflecting the higher cell density used in the suspension transfection.

Consequently, the calculated specific activity was significantly higher for vectors produced in adherent cells (41.6 ± 7.2 TU/pg p24) compared to those from suspension cultures (14.1 ± 3.4 TU/pg p24; *p* < 0.01). This 2.9-fold difference suggests that while the suspension system produces a greater absolute number of viral particles, a lower proportion of these particles are infectious, likely due to the increased production of non-infectious or defective particles at high cell densities.

To provide a clear overview of the differences between the two platforms, we compiled the key parameters into Table 3. Despite the lower specific activity, the suspension system enables a substantially higher total functional yield per batch (4.41–4.76 × 10^9^ TU) due to the drastically increased cell density and culture volume, all in a serum-free, scalable format.

### 3.7. Functional Activity of Lentiviral Vectors

To evaluate the biological activity of the LV/CAR vector, both production batches were used to transduce primary human T lymphocytes (CD4^+^ and CD8^+^) at a multiplicity of infection (MOI) of 2 TU/cell, as described in Section 2.15.

**Figure 6:**
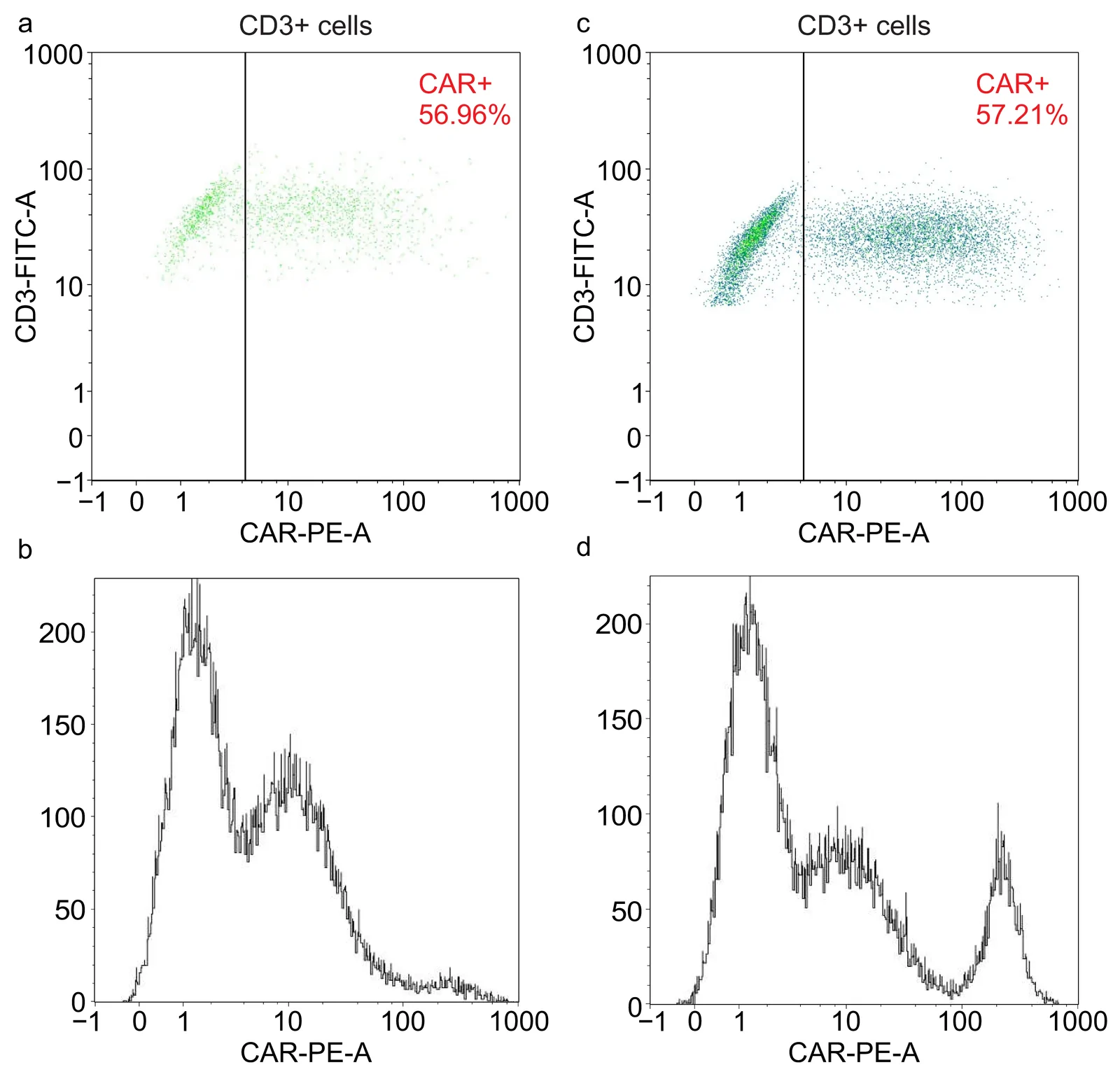
Functional validation of LV/CAR vectors in primary human T cells. T cells were transduced with vectors from two independent production batches (batch 1, **a**,**b**; batch 2, **c**,**d**) at an MOI of 2. CAR expression on viable CD3^+^ T cells was assessed by flow cytometry. (**a**,**c**) Representative dot plots showing CAR expression on the CD3^+^-gated population; the percentages of CAR^+^ cells are indicated. (**b**,**d**) Corresponding histograms showing the fluorescence intensity distribution of CAR staining.

Flow cytometric analysis of the transduced cells revealed CAR expression in 56.96% and 57.21% of viable CD3^+^ T cells for batches 1 and 2, respectively (Figure 6). Among CAR^+^ cells, the majority were CD4^+^ (62.33% and 56.72%) or CD8^+^ (33.91% and 41.64%). These two independent batches yielded highly reproducible transduction efficiencies, demonstrating the robustness of the production process. No CAR expression was detected in CD3^−^ populations, confirming the specificity of the CAR transgene for T cells.

Together, these results demonstrate that the LV/CAR vector produced in suspension-adapted HEK293FT-DS cells efficiently transduces primary human T cells, supporting its suitability for academic CAR-T cell manufacturing.

## 4. Discussion

The growing demand for clinical-grade vectors as gene delivery vehicles in the rapidly expanding field of cell and gene therapy (CGT) has mandated scalable, cost-effective vector production methods [4,8,9]. Although transient transfection of adherent HEK293 cells remains the most common approach in academic laboratories, its reliance on serum-containing media and multi-layer plastic vessels makes scale-up labour-intensive and expensive [7,10]. In this study, we developed an open-system alternative based on a newly generated suspension-adapted HEK293FT cell line, designated HEK293FT-DS, and an optimised high-density transfection protocol.

We adapted HEK293FT cells to serum-free FreeStyle 293 Expression Medium using two methods. Direct adaptation was faster than sequential adaptation and resulted in superior viability (>93% vs. 89.5%) and a higher specific growth rate (μ = 0.54 day^−1^, doubling time 31 h). These growth properties remained stable over 35 passages, indicating phenotypic stability essential for scaling LV production. The HEK293FT-DS cell line therefore compares favourably with other suspension-adapted HEK293 lines [10,24,25].

After adaptation to serum-free suspension culture, LV titers obtained with the PEI-based transfection protocol devised for adherent cells were approximately 50-fold lower, a reduction consistent with previous reports for cells grown in complex serum-free media [13,24]. Inspired by Backliwal et al. [26], who showed that increasing cell density during transfection could overcome medium-associated inhibition, we systematically raised the cell density during transfection. Increasing from 1 × 10^6^ to 16 × 10^6^ cells/mL boosted functional LV titers by more than two orders of magnitude, reaching approximately 9 × 10^6^ TU/mL in SFM. Further increases in cell density did not improve titers, possibly because LV assembly imposes higher metabolic demands than production of a soluble recombinant protein [26]. We therefore selected 16 × 10^6^ cells/mL as optimal.

The optimal PEI:DNA mass ratio for suspension cells was 2.5:1, lower than the 3:1 ratio found for adherent cells. This observation is supported by other studies that identified an optimal PEI:DNA ratio of 2:1 for transfecting suspension-adapted HEK-293 cells [13].

Using the optimised parameters, we performed four parallel transfections and produced 1 L of LV-containing supernatant. Two-step concentration by tangential flow filtration and ultracentrifugation gave a final functional yield of 64–71%, which compares favourably with the 55% recovery reported by Bauler et al. [10] and is generally higher than typical recoveries from adherent cell supernatants [7]. The final concentrated titers (3.15–3.40 × 10^8^ TU/mL) were sufficient for clinical-scale CAR-T cell production (e.g., sufficient to transduce above 10^8^ T-cells, a typical starting amount in the Miltenyi Biotec clinical CAR-T manufacturing process).

The specific activity of the unconcentrated suspension supernatant (14.1 ± 3.4 TU/pg) fell within the range of 10–50 TU/pg reported for crude HEK293-based LV supernatants [27,28]. Following tangential flow filtration and ultracentrifugation, the specific activities of the final concentrated batches increased to 856 and 997 TU/pg, respectively. This substantial increase over the crude supernatant value reflects the efficient removal of free p24 and non-infectious particles during the two-step concentration process.

Functional validation of the LV/CAR vector was performed by transducing primary human T cells (CD4^+^ and CD8^+^) at a moderate multiplicity of infection (MOI). Our two LV/CAR batches yielded 56.96% and 57.21% CAR-positive cells within the viable CD3^+^ T-cell population. These transduction efficiencies are similar to those reported by Tirapelle et al. [24], who used vectors produced in a comparable suspension-adapted HEK293T system.

Other groups have also developed approaches to overcome the limitations of adherent cultures for scaled LV production. Commercial systems such as LV-MAX can achieve titers up to 10^8^ TU/mL, but rely on proprietary transfection enhancers specifically designed for high-titer LV production [20]. Such proprietary systems require costly licensing agreements, placing them out of reach for many academic laboratories. The key distinction of our HEK293FT-DS cell line is that it was derived from HEK293FT cells, which express the SV40 large T antigen—a feature absent in HEK293F and Expi293 cells. This property may enhance plasmid replication and potentially increase LV yields. Our platform uses widely available, low-cost reagents (PEI (polyethylenimine, PEI MAX; Polysciences, Warrington, PA, USA) and FreeStyle 293 Expression Medium (Gibco, Grand Island, NY, USA)) and an in-house adapted cell line, making it an accessible and cost-effective alternative for academic and resource-limited settings. While our peak titers in clarified supernatant (~9 × 10^6^ TU/mL) are lower than those of LV-MAX, the overall yield of 4–5 × 10^9^ TU per batch is sufficient for clinical-scale CAR-T cell manufacturing.

Another strategy is the use of stable producer cell lines (PCLs), which eliminate the need for plasmid transfection altogether. While PCLs may be desirable in true industrial settings, their generation is time-consuming and labour-intensive because it requires multiple rounds of clonal selection and validation [8,11,12,25]. For academic laboratories that frequently work with different CAR constructs, a flexible transient transfection system based on an efficient, suspension-adapted cell line—such as the one described here—offers the best balance of speed, cost, and performance.

Several limitations of this study should be acknowledged. First, while we demonstrated stable growth over 35 passages, extended monitoring beyond 50 passages would be required for full master cell bank qualification. Second, the adaptation experiments were performed once; although subsequent stability data from three biological replicates confirm the robustness of the resulting cell line, independent adaptation events would further strengthen this conclusion. Third, scale-up was performed in shake flasks rather than in controlled stirred-tank bioreactors; evaluation under dynamic bioreactor conditions (pH, DO control) will be necessary for industrial implementation. Fourth, while we demonstrated efficient CAR expression in primary T cells, functional assays (cytotoxicity, cytokine release) were not performed, as the primary objective of this study was to establish the LV production platform. Finally, comprehensive QC metrics (HCP, hcDNA, RCL) were beyond the scope of this academic study but will be essential for clinical-grade manufacturing.

The described LV production platform, based on suspension adaptation and high-density transfection, provides a low-cost, open system well suited for academic use.

## 5. Conclusions

We generated a suspension-adapted HEK293FT cell line (HEK293FT-DS) that enables high-density transfection, providing a platform for producing lentiviral vectors for CAR-T therapy. The platform uses widely accessible materials and is easy to scale, achieving functional titers of approximately 7 × 10^6^ TU/mL in clarified supernatant and overall yields of 4–5 × 10^9^ TU after two-step concentration. These performance metrics demonstrate its practical utility for academic and decentralized manufacturing settings.

## Figures and Tables

**Table 1:** Growth parameters of HEK293FT-DS cells.

Parameter ^1^	Mean ± SD
Viable cell density	1.505 ± 0.028
Specific growth rate μ (day^−1^)	0.538 ± 0.006
Population doubling time Td (h)	30.95 ± 0.35

Comments: ^1^ Computed from three biological replicates (*n* = 3) for each of the 35 passages (combined 105 measurements).

**Table 2:** Scaled production of lentiviral vectors.

Process Stage	Final Volume (mL)	Concentration Factor (Fold)	Functional Titer (TU/mL) ^1^	Total Yield (TU) ^1^	Stage Yield (%) ^1^
Supernatant collection	1000	1×	6.87 × 10^6^ 6.71 × 10^6^	6.87 × 10^9^ 6.71 × 10^9^	100% ^2^ 100%
Concentration (TFF)	28	~35.7×	2.23 × 10^8^ 2.23 × 10^8^	6.23 × 10^9^ 6.25 × 10^9^	90.7% 93.1%
Purification by centrifugal pelleting	14	2×	3.15 × 10^8^ 3.40 × 10^8^	4.41 × 10^9^ 4.76 × 10^9^	70.8% 76.2%
Final yield	14	71.4×	3.15 × 10^8^ 3.40 × 10^8^	4.41 × 10^9^ 4.76 × 10^9^	64.2% 70.9%

Comments: ^1^ Data from two independently produced vector batches are presented; for each process stage, the upper value corresponds to batch 1 and the lower value to batch 2. ^2^ For the supernatant collection stage, the yield was arbitrarily set to 100%. Since only two large-scale batches were produced, no statistical analysis was performed on the production data.

**Table 3:** Comparative production parameters for adherent HEK293FT and suspension-adapted HEK293FT-DS cells.

Parameter	Adherent HEK293FT	Suspension HEK293FT-DS
Cell culture format	Adherent monolayer (static)	Suspension (shaker flask)
Culture medium	DMEM + 10% FBS	FreeStyle 293 Expression Medium (serum-free)
Transfection cell density	1 × 10^5^ cells/cm^2^	16 × 10^6^ cells/mL
Optimal PEI:DNA ratio	3:1	2.5:1
Functional titer in clarified supernatant	(1.29 ± 0.21) × 10^7^ TU/mL	(9.0 ± 1.5) × 10^6^ TU/mL
p24 antigen concentration in supernatant	318 ± 42 ng/mL	652 ± 89 ng/mL
Specific activity (TU/pg p24)	41.6 ± 7.2 TU/pg p24	14.1 ± 3.4 TU/pg p24
Production scale per batch	Limited by surface area	Shaker flasks (easily scalable)
Total yield after concentration (TFF + UC)	n.d.	(4.41–4.76) × 10^9^ TU
Overall recovery after concentration	n.d.	64–71%
Long-term phenotypic stability	n.d.	Stable over 35 passages (μ = 0.538 day^−1^, Td = 30.95 h)
CAR expression in primary T cells (MOI 2)	n.d.	~57% CAR^+^ (56.96–57.21%)

Comments: TU, transducing units; TFF, tangential flow filtration; UC, ultracentrifugation; n.d., not determined.

## Data Availability

Data is contained within the article or Supplementary Materials.

## Supplementary Materials

The following supporting information can be downloaded at: https://www.mdpi.com/article/10.3390/bioengineering13080856/s1.

## Abbreviations

The following abbreviations are used in this manuscript:

CAR	Chimeric antigen receptor
CGT	Cell and gene therapy
DMSO	Dimethyl sulfoxide
FBS	Fetal bovine serum
GFP	Green fluorescent protein
gRV	Gamma-retroviral vector
hcDNA	Host cell DNA
HCP	Host cell protein
HEPES	4-(2-hydroxyethyl)-1-piperazineethanesulfonic acid
HIV	Human immunodeficiency virus
HSA	Human serum albumin
IL-7	Interleukin-7
IL-15	Interleukin-15
LV	Lentiviral vector
MOI	Multiplicity of infection
NEAA	Non-essential amino acids
PBS	Phosphate-buffered saline
PCL	Packaging cell line
PE	Phycoerythrin
PEI	Polyethylenimine
PES	Polyethersulfone
QC	Quality control
RCL	Replication-competent lentivirus
RT	Room temperature
SD	Standard deviation
SFM	Serum-free medium
TFF	Tangential flow filtration
TU	Transducing units
VSV-G	Vesicular stomatitis virus G protein

## References

[1] Kuttiappan A., Chenchula S., Vardhan K.V., Padmavathi R., Varshini T.S., Amerneni L.S., Amerneni K.C., Chavan M.R. (2025). CAR T-cell therapy in hematologic and solid malignancies: Mechanisms, clinical applications, and future directions. Med. Oncol..

[2] June C.H., Sadelain M. (2018). Chimeric Antigen Receptor Therapy. N. Engl. J. Med..

[3] Bayat M., Nahand J.S. (2026). CAR-engineered cell therapies: Current understandings and future perspectives. Mol. Biomed..

[4] Vormittag P., Gunn R., Ghorashian S., Veraitch F.S. (2018). A guide to manufacturing CAR T cell therapies. Curr. Opin. Biotechnol..

[5] Naldini L., Blömer U., Gallay P., Ory D., Mulligan R., Gage F.H., Verma I.M., Trono D. (1996). In vivo gene delivery and stable transduction of nondividing cells by a lentiviral vector. Science.

[6] Milone M.C., O’Doherty U. (2018). Clinical use of lentiviral vectors. Leukemia.

[7] Segura M.M., Mangion M., Gaillet B., Garnier A. (2013). New developments in lentiviral vector design, production and purification. Expert Opin. Biol. Ther..

[8] Merten O.W., Hebben M., Bovolenta C. (2016). Production of lentiviral vectors. Mol. Ther. Methods Clin. Dev..

[9] van Heuvel Y., Stitz J. (2025). Development of Lentiviral Packaging Cells and Scale Up of Production to Meet the Growing Demand in Cell and Gene Therapy. Curr. Gene Ther..

[10] Bauler M., Roberts J.K., Wu C.C., Fan B., Ferrara F., Yip B.H., Diao S., Kim Y.-I., Moore J., Zhou S. (2019). Production of lentiviral vectors using suspension cells grown in serum-free media. Mol. Ther. Methods Clin. Dev..

[11] Tridgett M., Mulet M., Johny S.P., Ababi M., Raghunath M., Fustinoni C., Galabova B., Fernández-Díaz C., Mikalajūnaitė I., Tomás H.A. (2024). Lentiviral vector packaging and producer cell lines yield titers equivalent to the industry-standard four-plasmid process. Mol. Ther. Methods Clin. Dev..

[12] Tran M.Y., Dash S., Yang Z., Kamen A.A. (2023). Integrated Semi-Continuous Manufacturing of Lentiviral Vectors Using a HEK-293 Producer Cell Line. Processes.

[13] Ansorge S., Lanthier S., Transfiguracion J., Durocher Y., Henry O., Kamen A. (2009). Development of a scalable process for high-yield lentiviral vector production by transient transfection of HEK293 suspension cultures. J. Gene Med..

[14] Celebi Torabfam G., Yetisgin A.A., Erdem C., Cayli A., Kutlu O., Cetinel S. (2022). A feasibility study of different commercially available serum-free mediums to enhance lentivirus and adeno-associated virus production in HEK 293 suspension cells. Cytotechnology.

[15] Backliwal G., Hildinger M., Kuettel I., Delegrange F., Hacker D.L., Wurm F.M. (2008). Valproic acid: A viable alternative to sodium butyrate for enhancing protein expression in mammalian cell cultures. Biotechnol. Bioeng..

[16] Tom R., Bisson L., Durocher Y. (2008). Transfection of HEK293-EBNA1 Cells in Suspension with 293fectin for Production of Recombinant Proteins. CSH Protoc..

[17] Lee J.Y., Huh H.D., Lee D.K., Park S.Y., Shin J.E., Gee H.Y., Park H.W. (2024). Reprogramming anchorage dependency to develop cell lines for recombinant protein expression. Biotechnol. J..

[18] Lomba A.L.O., Tirapelle M.C., Biaggio R.T., Abreu-Neto M.S., Covas D.T., Picanço-Castro V., Swiech K., Mizukami A. (2021). Serum-Free Suspension Adaptation of HEK-293T Cells: Basis for Large-Scale Biopharmaceutical Production. Braz. Arch. Biol. Technol..

[19] Kuo H.-J., Lu M., Rungkittikhun C., Hu W.-S. (2025). Transcriptomic functional characterization of recombinant adeno-associated virus producing cell line adapted to suspension-growth. Biotechnol. Prog..

[20] Thermo Fisher Scientific LV-MAX™ Lentiviral Production System. Catalog No. A35684. https://www.thermofisher.com/order/catalog/product/A35684.

[21] Keyer V., Syzdykova L., Ingirbay B., Sedova E., Zauatbayeva G., Kulatay T., Shevtsov A., Shustov A.V. (2024). Non-industrial production of therapeutic lentiviral vectors: How to provide vectors to academic CAR-T. Biotechnol. Bioeng..

[22] Syzdykova L., Zauatbayeva G., Keyer V., Ramanculov Y., Arsienko R., Shustov A.V. (2023). Process for production of chimeric antigen receptor-transducing lentivirus particles using infection with replicon particles containing self-replicating RNAs. Biochem. Eng. J..

[23] Keyer V., Kydyrbayeva A., Kulatay T., Zauatbayeva G., Bazhenov D., Ingirbay B., Shakhmanova Z., Zhumabekova M., Ospanova M., Shustov A.V. (2025). Introducing CAR-T Therapy in Kazakhstan: Establishing Academic-Scale Lentiviral Vector and CAR-T Cell Production. Biomolecules.

[24] Tirapelle M.C., Oliveira Lomba A.L., Silvestre R.N., Mizukami A., Covas D.T., Picanço-Castro V., Swiech K. (2022). Transition from serum-supplemented monolayer to serum-free suspension lentiviral vector production for generation of chimeric antigen receptor T cells. Cytotherapy.

[25] Manceur A.P., Kim H., Misic V., Andreev N., Dorion-Thibaudeau J., Lanthier S., Bernier A., Tremblay S., Gélinas A.M., Broussau S. (2017). Scalable Lentiviral Vector Production Using Stable HEK293SF Producer Cell Lines. Hum. Gene Ther. Methods.

[26] Backliwal G., Hildinger M., Hasija V., Wurm F.M. (2008). High-density transfection with HEK-293 cells allows doubling of transient titers and removes need for a priori DNA complex formation with PEI. Biotechnol. Bioeng..

[27] Soldi M., Sergi L.S., Unali G., Kerzel T., Cuccovillo I., Capasso P., Annoni A., Biffi M., Rancoita P.M.V., Cantore A. (2020). Laboratory-Scale Lentiviral Vector Production and Purification for Enhanced Ex Vivo and In Vivo Genetic Engineering. Mol. Ther. Methods Clin. Dev..

[28] OriGene Technologies FAQs: Lentivirus and AAV—What Are the Limitations of Using P24 ELISA for Quantifying Lentivirus Titer?. https://www.origene.com/support/product-support/faqs?productLine=Lentivirus%20and%20AAV.

