# Supplementary material for: Suspension-Adapted HEK293FT Cells Enable High-Density Transfection for Efficient Lentiviral Vector Production in CAR-T Therapy
Source: Bioengineering (Basel). 2026 Jul 24;13(8):856. doi: 10.3390/bioengineering13080856 (PMC13510166; doi:10.3390/bioengineering13080856)
Supplement: Supplementary file 1 [file bioengineering-13-00856-s001.zip › bioengineering-4398325-supplementary.pdf]

Supplementary material to

Suspension-Adapted HEK293FT Cells Enable High-Density Transfection for Efficient Lentiviral Vector Production in CAR-T Therapy

Alexandr Shevtsov [1], Aitolkyn Kydyrbayeva [1], Gaziza Nigmatulla [1], Viktoriya Keyer [1], Tolganay Kulatay [1], Gulzat Zauatbayeva [1], Bakytkali Ingirbay [1], Maral Zhumabekova [1], Dinara Zharlyganova [1], Alexandr V. Shustov [1,*]

1 National Center for Biotechnology, Korgalzhin hwy 13/5, 010000, Astana, Kazakhstan

* Correspondence: shustov@biocenter.kz , Tel: +77024735305

A.S.	Alexandr Shevtsov	ORCID 0000-0002-0307-1053	shevtsov@biocenter.kz
A.K.	Aitolkyn Kydyrbayeva	ORCID 0009-0003-2199-5756	aitolkyn.yk@gmail.com
G.M.	Gaziza Nigmatulla	ORCID 0009-0001-7868-3396	nigmatullag@gmail.com
V.K.	Viktoriya Keyer	ORCID 0000-0001-8885-2387	keer@biocenter.kz
T.K.	Tolganay Kulatay	ORCID 0009-0004-5885-8963	kulatay@biocenter.kz
G.Z.	Gulzat Zauatbayeva	ORCID 0000-0003-1514-9302	zauatbaeva@biocenter.kz
B.I.	Bakytkali Ingirbay	ORCID 0000-0002-6915-8207	ingirbay@biocenter.kz
M.Z.	Maral Zhumabekova	ORCID 0009-0002-9532-6232	zhumabekova@biocenter.kz
D.Z.	Dinara Zharlyganova	ORCID 0000-0001-6383-4035	dinarazh@mail.ru
A.V.S.	Alexandr V. Shustov	ORCID 0000-0001-9880-9382	shustov@biocenter.kz

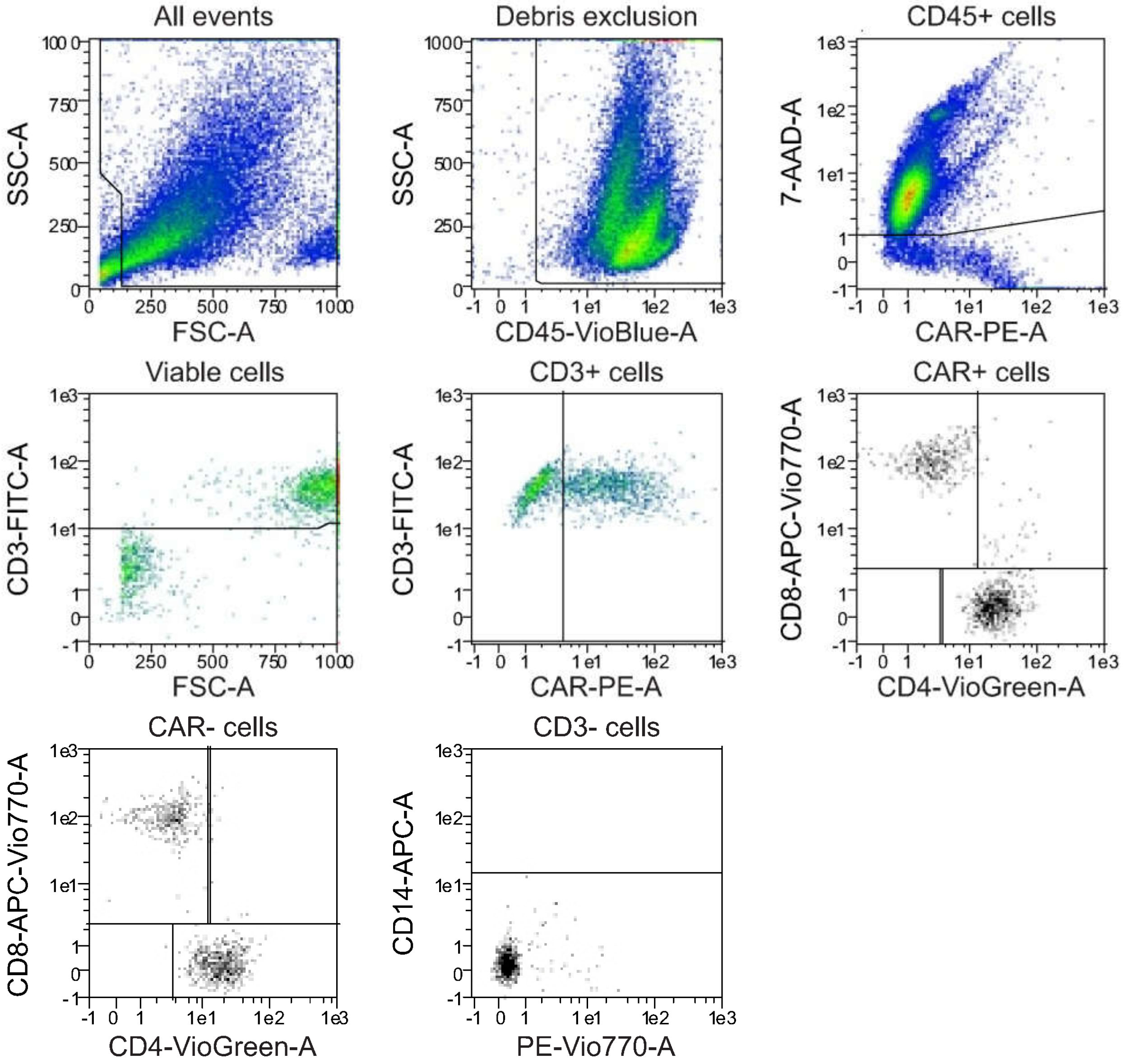

Figure S1. Characterization of cell types in CAR-T cell product #1 obtained in this study using vector batch #1. The vector was packaged in suspension-adapted HEK293FT-DS cells. Representative flow cytometry scatter plots show the distribution of cell populations by surface marker expression, including CAR expression. The gates used to isolate cell populations are indicated, and cell surface markers are labeled on the axes.

Cell type	Defined population	Count	Percent
Sample	All acquired events	29550	
Debris exclusion	FSC small events excluded	26249	88.83
CD45+ cells	FSC small events excluded and CD45+	26097	99.42
Viable CD45+ cells	CD45+ 7-AAD-	2199	8.43
CD3+ cells	Viable CD3+	1631	74.17
CAR+ cells	Viable CD3+ CAR+	929	56.96
CAR+ CD4+ cells	Viable CD3+ CAR+ CD4+ CD8-	579	62.33
CAR+ CD8+ cells	Viable CD3+ CAR+ CD4- CD8+	315	33.91
CAR+ CD4+ CD8+ cells	Viable CD3+ CAR+ CD4+ CD8+	35	3.77
CAR+ CD4- CD8- cells	Viable CD3+ CAR+ CD4- CD8-	0	0
CAR- cells	Viable CD3+ CAR-	702	43.04
CAR- CD4+ cells	Viable CD3+ CAR- CD4+ CD8-	423	60.26
CAR- CD8+ cells	Viable CD3+ CAR- CD4- CD8+	270	38.46
CAR- CD4+ CD8+ cells	Viable CD3+ CAR- CD4+ CD8+	8	1.14
CAR- CD4- CD8- cells	Viable CD3+ CAR- CD4- CD8-	1	0.14
CD3- cells	Viable CD3-	568	25.83
Monocytes	Viable CD3- CD14+ among CD45+ 7-AAD-	0	0

CAR+ cells in viable CD3+	CD3+ CAR+ in CD45+ 7-AAD- CD3+	929	56.96
CAR+ cells in viable cells	CD3+ CAR+ in CD45+ 7-AAD-	929	42.25

Table S1. Summary table of cell type representation in CAR-T cell product #1 obtained in this study using vector batch #1. The vector was packaged in suspension-adapted HEK293FT-DS cells. Shown are cell type, gating strategy, event count, and percentage.

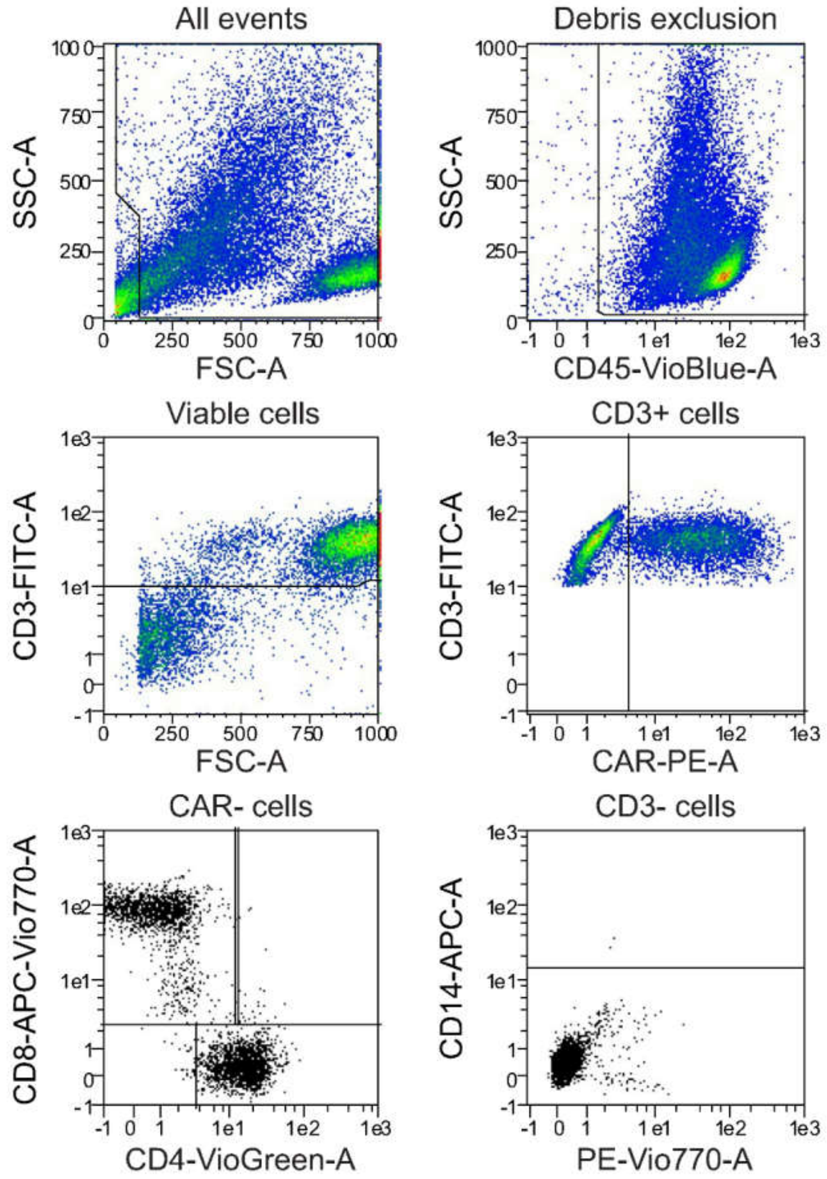

Figure S2. Characterization of cell types in CAR-T cell product #2 obtained in this study using vector batch #2. The vector was packaged in suspension-adapted HEK293FT-DS cells. Representative flow cytometry scatter plots show the distribution of cell populations by surface marker expression, including CAR expression. The gates used to isolate cell populations are indicated, and cell surface markers are labeled on the axes.

Cell type	Defined population	Count	Percent
Sample	All acquired events	27072	
Debris exclusion	FSC small events excluded	23898	88.28
CD45+ cells	FSC small events excluded and CD45+	23171	96.96
Viable CD45+ cells	CD45+ 7-AAD-	11308	48.80
CD3+ cells	Viable CD3+	8219	72.68
CAR+ cells	Viable CD3+ CAR+	4702	57.21
CAR+ CD4+ cells	Viable CD3+ CAR+ CD4+ CD8-	2667	56.72
CAR+ CD8+ cells	Viable CD3+ CAR+ CD4- CD8+	1958	41.64
CAR+ CD4+ CD8+ cells	Viable CD3+ CAR+ CD4+ CD8+	77	1.64
CAR+ CD4- CD8- cells	Viable CD3+ CAR+ CD4- CD8-	0	0
CAR- cells	Viable CD3+ CAR-	3517	42.79
CAR- CD4+ cells	Viable CD3+ CAR- CD4+ CD8-	1843	52.40
CAR- CD8+ cells	Viable CD3+ CAR- CD4- CD8+	1609	45.75
CAR- CD4+ CD8+ cells	Viable CD3+ CAR- CD4+ CD8+	27	0.77
CAR- CD4- CD8- cells	Viable CD3+ CAR- CD4- CD8-	38	1.08
CD3- cells	Viable CD3-	3089	27.32
Monocytes	Viable CD3- CD14+ among CD45+ 7-AAD-	2	0.02

CAR+ cells in viable CD3+	CD3+ CAR+ in CD45+ 7-AAD- CD3+	4702	57.21
CAR+ cells in viable cells	CD3+ CAR+ in CD45+ 7-AAD-	4702	41.58

Table S2. Summary table of cell type representation in CAR-T cell product #2 obtained in this study using vector batch #2. The vector was packaged in suspension-adapted HEK293FT-DS cells. Shown are cell type, gating strategy, event count, and percentage.
